# Effects of grape seed proanthocyanidin extract on pentylenetetrazole-induced kindling and associated cognitive impairment in rats

**DOI:** 10.3892/ijmm.2014.1796

**Published:** 2014-06-06

**Authors:** JUNLI ZHEN, ZHENZHEN QU, HAIBO FANG, LAN FU, YUPENG WU, HONGCHAO WANG, HONGMIN ZANG, WEIPING WANG

**Affiliations:** Department of Neurology, Key Laboratory of Neurology of Hebei Province, The Second Hospital of Hebei Medical University, Shijiazhuang, Hebei 050000, P.R. China

**Keywords:** cognitive impairment, proanthocyanidins, pentylenetetrazole, morris water maze, apoptosis, oxidative stress

## Abstract

Numerous studies have demonstrated the antioxidant effects of grape seed proanthocyanidin extract (GSPE). The generation of free radicals and the ensuing apoptosis may contribute to the pathogenesis of epilepsy; therefore, in the present study, we examined the effects of GSPE on cognitive impairment and neuronal damage induced by chronic seizures in rats. Seizures were induced by a daily intraperitoneal (i.p.) injection of pentylenetetrazole (PTZ; 35 mg/kg/day, 36 days). Two other groups were treated with GSPE (100 or 200 mg/kg/day, orally) for 24 days and then for 36 days prior to each PTZ injection. After the final PTZ injection, hippocampus-dependent spatial learning was assessed using the Morris water maze (MWM). The rats were then sacrificed for the measurement of hippocampal malondialdehyde (MDA, a measure of lipid peroxidation) and glutathione (GSH, a measure of endogenous antioxidant capacity) levels, and for the expression of pro-apoptotic factors [cytochrome *c* (Cyt *c*), caspase-9 and caspase-3]. The mitochondrial generation of reactive oxygen species (ROS), degree of mitochondrial swelling, neuronal damage and mitochondrial ultrastructure were also examined. Performance in the MWM was markedly impaired by PTZ-induced seizures, as evidenced by longer escape latencies during training and fewer platform crossings during the probe trial. This cognitive decline was accompanied by oxidative stress (MDA accumulation, ROS generation, reduced GSH activity), an increased expression of pro-apoptotic proteins, as well as damage to CA1 pyramidal neurons and the mitochondria. Pre-treatment with GSPE dose-dependently reversed PTZ-induced impaired performance in the MWM, oxidative stress, mitochondrial ROS generation, the expression of pro-apoptotic proteins and neuronal and mitochondrial damage. Thus, GSPE may reverse the hippocampal dysfunction induced by chronic seizures, by reducing oxidative stress and preserving mitochondrial function.

## Introduction

Epilepsy is a common, often intractable and occasionally fatal neurological disease. Many of the sequelae of chronic seizures result from excitotoxicity and oxidative stress with ensuing mitochondrial damage and neuronal death (1,2). Although there are numerous anti-epileptic drugs (AEDs) available for the management of seizures, they merely treat symptoms, rather than cure the disease and have side-effects that diminish the quality of life, including cognitive impairment (3,4).

Oxidative stress associated with seizure activity can greatly reduce mitochondrial function and is widely accepted as a contributor to learning and memory deficits resulting from epileptic seizures (5). Compared to other organs, the brain is extremely susceptible to damage by free radicals due to its relatively high levels of oxidative metabolism and comparatively low levels of both free-radical scavenging enzymes and antioxidant molecules (6). Glutathione (GSH) plays an important role in antioxidant defense in mammalian tissues (7,8); it has been shown to prevent ischemia-induced neuronal death and improve memory following an ischemic insult (9). The accumulation of malondialdehyde (MDA), an end product of lipid peroxidation, reflects the extent of oxidative stress and indirectly that of cellular antioxidant capacity (10,11). Mitochondrial oxidative stress and dysfunction not only result from seizures, but may directly contribute to epileptogenesis (12). High concentrations of reactive oxygen species (ROS) cause lipid peroxidation and damage to cell membranes, proteins and mitochondrial DNA (13), which exacerbates mitochondrial dysfunction, reduces mitochondrial energy production (14) and triggers apoptotic cell death through the activation of the caspase-3 pathway (3,15). Hence, exogenous antioxidants may be safe and effective agents for seizure control and the prevention of cognitive decline.

Dietary antioxidants may preserve mitochondrial function and energy metabolism, and have other long-term therapeutic benefits (16). The natural antioxidant, grape seed proanthocyanidin extract (GSPE), an extract from red grape seeds containing a variety of phenolic compounds, is widely marketed in China as a dietary supplement with a variety of health benefits. It is water-soluble and can cross the blood-brain barrier more easily than other natural antioxidants, such as quercetin (17) and curcumin (18). In addition, GSPE has demonstrated a wide range of biological effects, including antioxidant (19,20), anti-inflammatory (21,22), anti-mutagenic (23), anti-carcinogenic (24), cardioprotective (25,26) and neuroprotective (27) activities in various experimental models; furthermore, it can effectively reduce ischemia/reperfusion injury (28,29). Moreover, it has been reported to enhance working memory, ameliorate symptoms of Alzheimer’s disease (30), and significantly improve cognitive performance across age groups (31). However, little is known about the potential neuroprotective efficacy of GSPE against pentylenetetrazole (PTZ)-induced seizures.

The aim of the present study was to evaluate the protective effects of GSPE against seizure-induced cognitive impairment, oxidative stress caused by mitochondrial damage and neuronal apoptosis mediated by the caspase-3 signaling pathway. Our results revealed that GSPE effectively suppressed oxidative stress and neuronal apoptosis induced by epileptic seizures, and improved cognitive decline in epileptic rats.

## Materials and methods

### Animals

Ninety male Sprague-Dawley rats (180–200 g) were obtained from the Experimental Animal Center of Hebei Medical University. [Animal licenses: SCXK (Hebei 2008-1-003); animal certification no. 1301020]. The animals were allowed to acclimatize for 1 week prior to the experiments. The animals were housed in groups of 4 per cage in a room that was maintained under standard laboratory conditions (12 h light/dark cycle) and a constant temperature (25±1°C) and humidity (50–60%). They were allowed free access to food and water. All experiments conformed to the National Institutes of Health guidelines for the Care and Use of Laboratory Animals and with the European Communities Council Directive of 24 November 1986 (86/609/EEC). Every effort was made to minimize the number of animals used and their suffering.

### Materials

GSPE (purity >98%) was kindly provided by Tianjin Jianfeng Natural Product R&D Co., Ltd. (Tianjin, China). The GABA(A)-receptor antagonist, PTZ (CAS:54-95-5; Sigma, St. Louis, MO, USA), was used for epileptic kindling to establish a chronic epileptic rat model. The levels of MDA and GSH were measured using ELISA kits (Nanjing Jiancheng Bioengineering Institute, Nanjing, China). Mitochondrial ROS production was measured by flow cytometry (BDFACSAria™ II; BD Biosciences, San Jose, CA, USA) using the oxidation-sensitive fluorescent dye, 2′,7′-dichlorodihydrofluorescein diacetate (DCFH-DA, CAS:D6883, Sigma). The animal model was established by an intraperitoneal (i.p.) injection of PTZ (35 mg/kg/day) for 36 days. GSPE and 1% PTZ were dissolved in physiological saline prior to the experiments. Drugs were administered between 08:30 and 10:30 a.m. to reduce the effects of circadian rhythms.

### Experimental groups and drug administration

The rats were randomly divided into 5 groups: i) a control group (n=16) that received normal saline (vehicle) for 60 days (3.5 ml/kg/day, i.p.); ii) a PTZ group (n=22) that received saline (3.5 ml/kg/day, i.p.) for 24 days followed by PTZ (35 mg/kg/day, i.p.) for 36 days; iii and iv) the PTZ + GSPE groups (n=18/group) that were treated with GSPE (100 or 200 mg/kg/day, by gavage) for 24 days then pre-treated with the same doses 30 min prior to each PTZ administration for 36 days; v) a GSPE alone group (n=16) that received 200 mg/kg/day GSPE only for 60 consecutive days. Following drug treatments, the mortality rate and seizure stages of the animals were recorded. Six rats per treatment group were tested in the Morris water maze (MWM) and were then sacrificed. The sections of the hippocampus were divided into 2 parts for the determination of oxidative stress and the measurement of the expression of pro-apoptotic proteins by western blot analysis (n=6/group). The remaining rats in each group were sacrificed and hippocampal tissue was processed for Nissl staining (n=3/group), the ultrastructural analysis of mitochondria by transmission electron microscopy (n=3/group) or assays of mitochondrial ROS generation and the degree of mitochondrial swelling (n=4/group).

### Behavioral assessment

The animals were observed for 1 h after each PTZ administration for changes in the seizure stage, which was assessed using the Racine scale: stage 0: no response; stage 1: hyperactivity, vibrissae twitching; stage 2: head nodding, clonus and myoclonic jerks of the head; stage 3: unilateral forelimb clonus; stage 4: rearing with bilateral forelimb clonus; stage 5: the generalized tonic-clonic seizure with loss of writing reflex.

### Behavioral screening

For behavioral screening, some rats (n=6/group) were tested in the MWM following the protocols previously described (37). The MWM consisted of a circular water tank (180 cm in diameter, 70 cm in height) filled with 22±1°C water. The pool was divided into 4 equal quadrants labeled N, W, S and E. A colorless escape platform (10 cm in diameter) was submerged 2 cm below the surface. Training was performed in 2 sessions daily for 5 days with an inter-session interval of 2 h. Each session consisted of 4 trials with an inter-trial interval of 30 sec. In each trial, the rat was gently placed in a randomly selected quadrant with its nose pointing toward the wall and allowed to find the escape platform. If a rat failed to find the platform within 120 sec, the escape latency was recorded as 120 sec and the rat was placed on the platform. The rats remained on the platform for 10 sec before the start of the next trial. On the 6th day, a probe trial of spatial memory was conducted by removing the platform and measuring both the time spent in the target quadrant and the number of crossings over the former platform location. The MWM was conducted between 9:00 a.m. and 18:00 p.m. to minimize performance variations due to circadian rhythmicity.

### Nissl staining

Fifteen animals (n=3/group) were decapitated and a 5-mm-thick coronal section, including the bilateral hippocampus, was excised from the brains. The sections were fixed in 4% paraformaldehyde for 24 h, dehydrated in alcohol, cleared with xylene and embedded in paraffin. The paraffin-embedded brain sections were sliced at 5 μm thickness and Nissl-stained with 1% thionin. In every 5th slice (3 slices per animal), we counted the number of surviving intact pyramidal cells per mm length of hippocampal CA1 subfield in both hemispheres. Neuronal cells were counted by 2 observers blinded to the treatment history using high magnification (×400) light microscopy.

### Transmission electron microscopy

Fifteen rats (n=3/group) were deeply anesthetized and processed for electron microscopy according to the method described in the study by Xie *et al* (32). Mitochondrial ultrastructure in the CA1 subfield was imaged (×40,000) using a transmission electron microscope (JEM-1230; Jeol Ltd., Tokyo, Japan) from 5 micrographs per rat.

### Measurement of MDA accumulation

The day after the completion of the behavioral tests, 30 rats (n=6/group) were sacrificed and the hippocampi were quickly removed and flash-frozen. The samples were thawed and homogenized in 1:9 w/v ice-cold normal saline. The homogenates were centrifuged at 3,000 × g for 15 min at 4°C and the supernatants were used for the determination of the MDA and GSH levels. The MDA concentration was measured according to the method described in the study by Ohkawa *et al* (33). Briefly, the substrate-supernatant mixture was centrifuged at 3,500 × g for 10 min, and the absorbance recorded at 532 nm using a spectrophotometer. The results were expressed in nmol MDA/g protein.

### Determination of GSH levels

GSH levels were measured using the method described in the study by Ellman (34). The substrate-supernatant mixture was vortexed and the absorbance read at 412 nm within 15 min. The GSH concentration was expressed in g GSH/g protein.

### Determination of mitochondrial ROS production and degree of mitochondrial swelling

After the final injection of PTZ, 20 rats (n=4/group) were sacrificed and the bilateral hippocampus was quickly removed and divided into 2 sections. Some samples were homogenized and the fluorescence intensity was immediately determined by flow cytometry at 488 nm excitation and 530 nm emission. Data were analyzed using BDFACSAria™ II Cell Sorter software (Version 7.0; BD Biosciences). Other samples were used to detect the degree of mitochondrial swelling by measuring the decrease in optical density at 520 nm, as previously described (35). The turbidity of the reaction mixture reflected the degree of mitochondrial swelling. Freshly prepared rat brain mitochondria (50 μg protein) were recorded over a period of 10 min at 25°C in 200 μl medium containing 250 mM sucrose, 5 mM KH_2_PO_4_ and 3 mM sodium succinate (pH 7.2).

### Western blot analysis

The hippocampi were collected and added to RIPA buffer with 1% PMSF and then lysed on ice. Total proteins were extracted (Applygen Technologies, Inc., Beijing, China) following the manufacturer’s instructions. Brain mitochondria isolation was conducted as previously described (35). The rat brain homogenate was centrifuged at 1,000 × g for 10 min, and the resulting supernatant was subjected to 10,000 × g centrifugation for 10 min. The pellet was the mitochondrial fraction. The supernatant was recentrifuged at 100,000 × g for 1 h at 4°C. The resulting supernatant was used as a cytosolic fraction. After determining protein concentrations, the proteins in the pellet and supernatant were loaded on 10% sodium dodecyl sulfate-polyacrylamide gels and transferred onto PVDF membranes (Millipore Corp., Bedford, MA, USA). The membranes were blocked for 1 h at room temperature and then incubated overnight at 4°C with one of the following primary antibodies: anti-*cytochrome c (*Cyt *c)* polyclonal antibody (pAb; 1:800, no. 4272; Cell Signaling Technology, Inc., Danvers, MA, USA), anti-caspase-9 pAb (1:500; sc-8355, Santa Cruz Biotechnology, Santa Cruz, CA, USA) and anti-caspase-3 pAb (1:600, bs-6428; Bioworld Technology, St. Louis Park, MN, USA) and β-actin (1:500, sc-47778; Santa Cruz Biotechnology). After washing them 3 times, the membranes were incubated with the appropriate HRP-conjugated secondary antibodies goat anti-rabbit (1:6,000; Rockland, Gilbertsville, PA, USA) at room temperature for 1 h. Following washing three times in TPBS and once in PBS alone, the immunolabeled marker protein bands on the membranes were scanned and analyzed (Odyssey; LI-COR, Inc., Lincoln, NE, USA). The densities of the Cyt *c*, caspase-9 and caspase-3 protein bands were measured and expressed as a ratio of the β-actin density.

### Statistical analysis

All data are expressed as the means ± SEM. Significance of the seizure stage was analyzed using a two-way analysis of variance (ANOVA). Escape latencies in the MWM were analyzed with repeated measures and multivariate ANOVA. Other data were compared by one-way ANOVA followed by the least significant difference (LSD) or Student-Newman-Keuls (SNK) post-hoc tests for multiple pair-wise comparisons. All statistical tests were calculated using SPSS 13.0 software (SPSS, Inc., Chicago, IL, USA) and P-values <0.05 were considered to indicate statistically significant differences.

## Results

### Behavioral assessment

**Figure 1:**
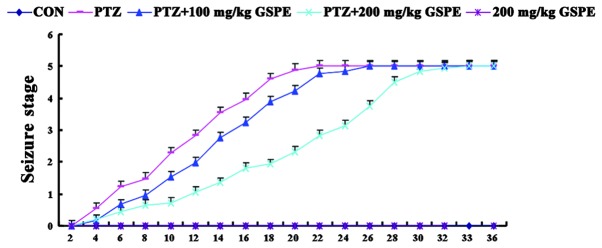
Effect of grape seed proanthocyanidin extract (GSPE) pre-treatment on behavior in rats with pentylenetetrazole (PTZ)-induced seizures. All animals showed a progressive aggravation of seizures after the injection with PTZ. The aggravation of seizures in the PTZ group was more marked and more rapid than in the other groups. These abnormal results were effectively reversed by high-dose GSPE pre-treatment. Values are expressed as the means ± SEM.

The injection of PTZ alone for 36 consecutive days caused significant mortality (27.3%), which was reduced by pre-treatment with 100 mg/kg GSPE (11.1%) and 200 mg/kg GSPE (5.6%). No fatalities occurred in the control group or the 200 mg/kg GSPE group, indicating that this high dosage has little or no inherent toxicity. As shown in Fig. 1, there was no seizure behavior in the control group and the 200 mg/kg GSPE group. On the other hand, severe epileptic seizures were induced by the kindling injections of PTZ for 36 days, and the PTZ group had epileptic seizures earlier than the other groups, while high-dose GSPE pre-treatment (200 mg/kg) significantly delayed the time to seizure. Although the rats in the low-dose group (100 mg/kg) exhibited mildly delayed seizures, the difference was not significant compared to the PTZ group (P=0.71), suggesting that GSPE may partially suppress epileptic seizures.

### GSPE reverses cognitive impairment following seizures

**Figure 2:**
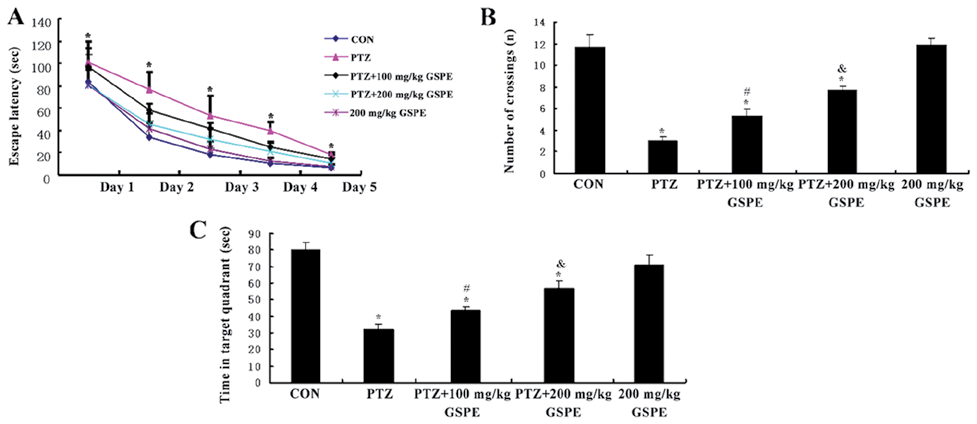
Effect of grape seed proanthocyanidin extract (GSPE) pre-treatment on performance in the Morris water maze (MWM) in pentylenetetrazole (PTZ)-treated epileptic rats. (A) Escape latencies in MWM training trials. (B) Number of platform crossings during the probe trial. (C) Time spent in the target quadrant. Values are expressed as the means ± SEM (n=6/group). ^*^P<0.05 compared to the control (CON) group. ^#^P<0.05, ^&^P<0.01 compared to the PTZ group.

To determine whether GSPE alleviates PTZ-induced cognitive impairment, we tested spatial learning and memory in the MWM following drug treatments (Fig. 2). The rats from all the groups showed a progressive decline in the escape latency during training (Fig. 2A), and this decline was more significant as the training days progressed [F(1,25)=928.84, P<0.001] and drug treatment group [F(4,25)=13.469, P<0.001]. The rats in the PTZ alone group (35 mg/kg/d) exhibited significantly prolonged escape latencies during all sessions compared to the control rats (P<0.001), while pre-treatment with GSPE dose-dependently decreased escape latency compared to the PTZ group (P=0.021 at 100 mg/kg and P<0.001 at 200 mg/kg). Escape latencies did not differ significantly between the high-dose GSPE group and the control group (P=0.570), indicating the near complete reversal of the PTZ-induced spatial learning deficit. In the probe trail (Fig. 2B and C), the rats treated with the vehicle + PTZ crossed the platform location significantly fewer times than the controls [F(4,25)=30.223, P<0.001] and spent less time in the target (former platform) quadrant than the control rats [F(4,25)=23.482, P<0.001]. The PTZ + GSPE groups also crossed the former platform location more often than the PTZ group and spent significantly more time in the target quadrant (P=0.045, P<0.001). Again, there was no significant difference in mean latency or the time in the target quadrant between the high-dose GSPE group and the control group (P=0.869, P=0.128), indicating the near complete reversal of the PTZ-induced spatial memory deficit.

### GSPE rescues hippocampal CA1 neurons

**Figure 3:**
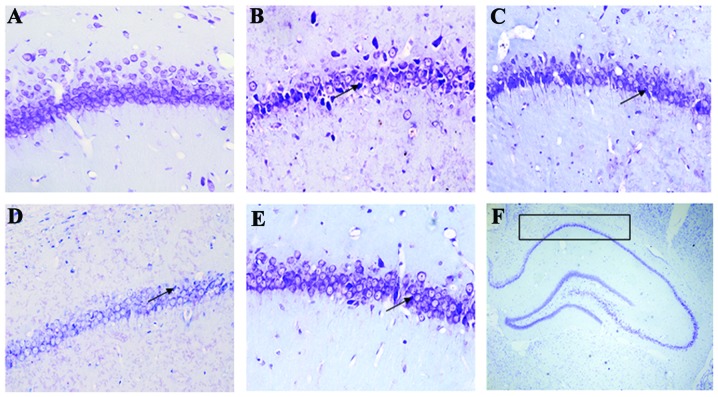
Grape seed proanthocyanidin extract (GSPE) rescues CA1 pyramidal neurons from seizure-induced damage as revealed by Nissl staining. (A) Control group; (B) pentylenetetrazole (PTZ) group; (C) PTZ + 100 mg/kg GSPE group; (D) PTZ + 200 mg/kg GSPE group; (E) GSPE alone group. Photomicrographs showing sample CA1 subfields (magnification, ×400) in the coronal plane for each treatment group. A damaged cell body is indicated by the black arrow. These signs of neural damage were reduced by GSPE pre-treatment. (F) Photomicrograph shows the whole hippocampal sample (magnification, ×40) in the coronal plane. CA1 subfield is indicated by the black frame. n=3/group.

**Figure 4:**
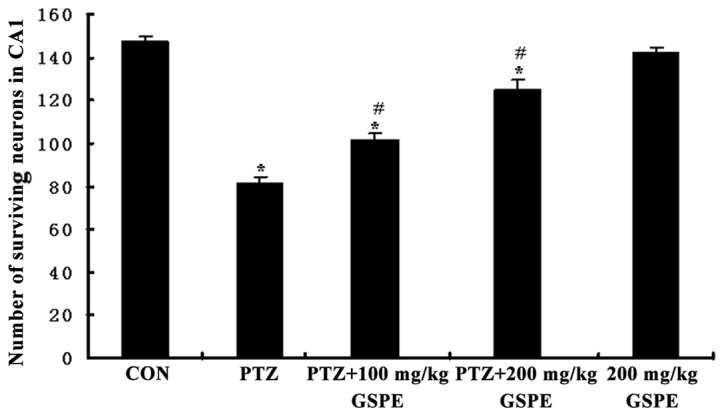
Effect of grape seed proanthocyanidin extract (GSPE) pre-treatment on the quantity of Nissl-positive CA1 pyramidal neurons in the hippocampus. The number of surviving pyramidal cells was counted under a light microscope and expressed as cells per mm linear length of the rat CA1 subfield. Data are shown as the means ± SEM. ^*^P<0.05 compared to the control (CON) group; ^#^P<0.05 compared to the pentylenetetrazole (PTZ) group.

Pyramidal neurons in the Nissl-stained sections from the control rats were evenly stained light blue and had regularly shaped cell bodies. By contrast, morphological abnormalities, including blurred caryotheca and pyknotic nuclei were apparent in sections from PTZ group rats. The average density of intact surviving neurons was lower in the PTZ group compared to the control group, while pre-treatment with GSPE reversed the damage to cell morphology (Fig. 3) and reversed the decline in CA1 pyramidal cell density (Fig. 4). Compared to the PTZ group, the number of damaged neurons was significantly and dose-dependently reduced by GSPE pre-treatment (P<0.05 for both low and high doses). The GSPE alone group showed comparable cell density to the control group, underscoring the low or absent neurotoxicity of the extract (P=0.254).

### GSPE preserves mitochondrial ultrastructure during seizures

**Figure 5:**
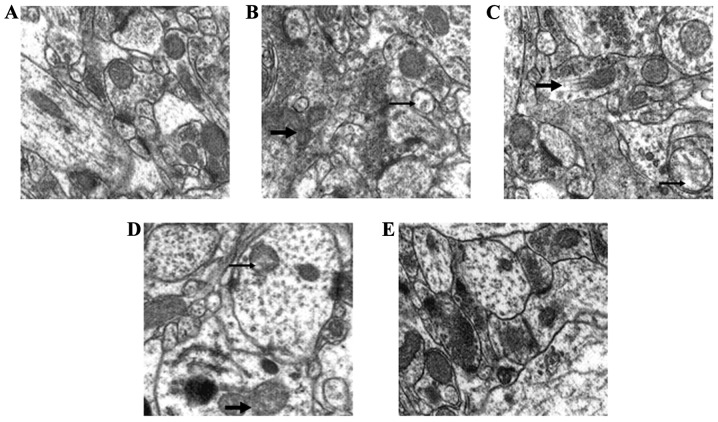
Pre-treatment with grape seed proanthocyanidin extract (GSPE) reverses pentylenetetrazole (PTZ)-induced ultrastructural damage to mitochondria. Photomicrographs (magnification, ×40,000) showing mitochondrial ultrastructure in the CA1 area of the hippocampus. (A) Control group; (B) PTZ group; (C) PTZ + 100 mg/kg GSPE group; (D) PTZ + 200 mg/kg GSPE group; (E) GSPE alone group. In (B), a mitochondrial vacuole is indicated by a thin black arrow and one damaged mitochondria by a thick black arrow. In the PTZ group, mitochondria ridge disorder and vacuoles were commonly observed. These abnormal changes were reversed by GSPE pre-treatment (C and D). n=3/group.

To demonstrate the effects of GSPE pre-treatment on the mitochondria, the morphology of the mitochondria was further observed. Compared to the control group, obvious morphological changes were observed in the mitochondria from the PTZ group, such as damaged membranes and obscure boundaries, whereas GSPE pre-treatment partly reversed these changes (Fig. 5).

### GSPE reduces MDA accumulation and upregulates GSH levels

**Figure 6:**
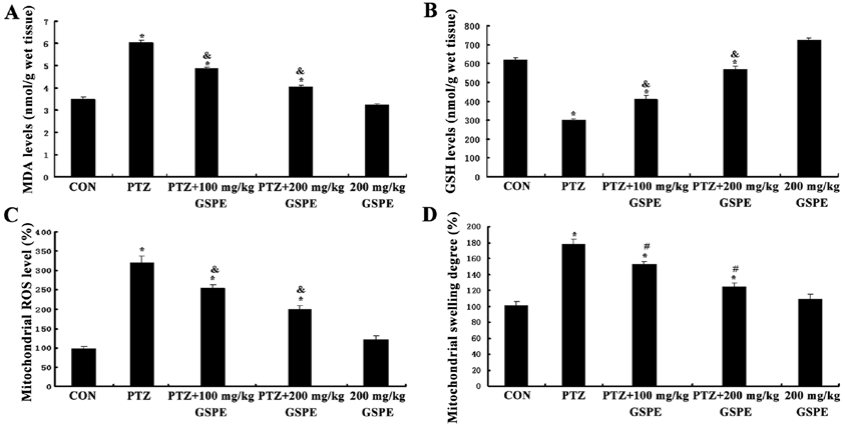
Effects of seizures and grape seed proanthocyanidin extract (GSPE) pre-treatment on the levels of malondialdehyde (MDA), glutathione (GSH) and mitochondrial reactive oxygen species (ROS) generation in the hippocampus. (A) MDA accumulation was increased by pentylenetetrazole (PTZ) and reduced by GSPE pre-treatment. (B) GSH was reduced by PTZ and restored by GSPE pre-treatment. (C) Mitochondrial ROS production, as detected by DCFH-DA fluorescence by flow cytometry, was higher in hippocampal tissue from the PTZ-treated rats and reversed by GSPE pre-treatment. (D) The degree of mitochondrial swelling was also higher in the PTZ-treated rats and markedly suppressed by GSPE pre-treatment. Bars represent the mean ± SEM (n=6/group in MDA and GSH, n=4/group in ROS and degree of mitochondrial swelling). ^*^P<0.05 compared to the control (CON) group; ^#^P<0.05, ^&^P<0.01 compared to the PTZ group.

As shown in Fig. 6A and B, there were significant group differences in both hippocampal MDA accumulation and GSH levels [F(4,25)=178.732, F(4,25)=518.96, P<0.001]. Compared to the control group, the accumulation of MDA was significantly higher (P<0.001) and GSH levels were lower in the PTZ group (P<0.001). Pre-treatment with GSPE dose-dependently reduced MDA and upregulated GSH levels compared to the PTZ group. Therefore, GSPE pre-treatment partially alleviated the oxidative stress induced by PTZ-induced seizures, although MDA levels were still higher and GSH levels still lower than the control group (P<0.05, P<0.05). There were no significant differences between the GSPE alone group and the control group (P=0.035, P=0.393).

### GSPE supresses PTZ-induced mitochondrial ROS production and degree of mitochondrial swelling

As shown in Fig. 6C, mitochondrial ROS generation was significantly higher in the PTZ group than the control group [F(4,15)=66.308, P<0.001], whereas pre-treatment with GSPE dose-dependently decreased mitochondrial ROS production compared to the PTZ group (P=0.001, P=0.003). Thus, GSPE partially suppressed mitochondrial ROS production in the hippocampus, although ROS levels were still higher than those in the control group. There was no significant difference in mitochondrial ROS production between the GSPE alone and control groups (P=0.18). Accordingly, mitochondrial swelling in the isolated brain mitochondria was monitored by measuring the change in absorbance of the suspension at 520 nm. As shown in Fig. 6D, the degree of mitochondrial swelling was significantly increased by 42.98% (P<0.001) in the PTZ group compared to the control group. Compared with the PTZ group, GSPE pre-treatment dose-dependently inhibited mitochondrial swelling by 29.8 and 14.4% (P<0.05 for both doses), respectively.

### GSPE inhibits mitochondrial Cyt c release induced by seizures

**Figure 7:**
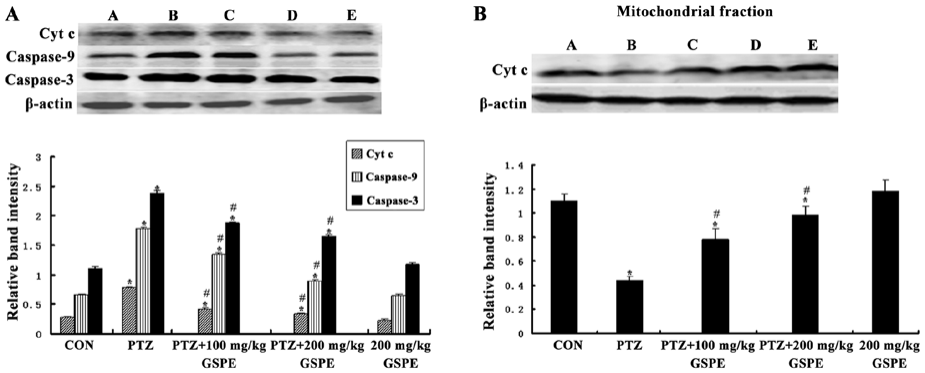
Effects of grape seed proanthocyanidin extract (GSPE) pre-treatment on the expression of apoptosis-related proteins in the hippocampus. (A) Control group; (B) pentylenetetrazole (PTZ) group; (C) PTZ + 100 mg/kg GSPE group; (D) PTZ + 200 mg/kg GSPE group; E) GSPE alone group. Relative densities of cytochrome *c* (Cyt *c*)/β-actin, caspase-9/β-actin and caspase-3/β-actin are shown in cytosolic fraction (A); relative densities of Cyt *c*/β-actin are shown in mitochondrial fraction (B). Bars represent the mean ± SEM (n=6/group). ^*^P<0.05 compared to the control (CON) group; ^#^P<0.05 compared to the PTZ group.

The translocation of mitochondrial Cyt *c* to the cytosol induces the formation of the apoptosomes, leading to apoptotic death. As shown in Fig. 7, there was a significant group difference in the level of cytosolic Cyt *c* [F(4,25)=141.021, P<0.001] and mitochondrial Cyt *c* [F(4,25)=234.398, P<0.001]. Compared to the control group, the cytoplasmic Cyt *c* concentration was markedly higher and the mitochondrial Cyt *c* concentration was markedly lower in the PTZ group (P<0.001, P<0.001), while pre-treatment with GSPE dose-dependently reversed those levels compared to the PTZ group (P<0.05 for both doses). There was no significant difference between the GSPE alone and control groups (P=0.096, P=0.632), indicating that high-dose GSPE has no endogenous pro-apoptotic activity.

### GSPE suppresses the PTZ-induced increase of in caspase-9 and caspase-3 expression

Caspase-3 is the principle effector of the mitochondrial-dependent apoptotic pathway and is activated during seizure-induced neuronal death (36). Western blot analysis revealed a significant group difference in both activated caspase-9 and caspase-3 expression in the hippocampus [F(4,25)=554.387, P<0.001; F(4,25)=347.38, P<0.001]. Compared to the control group, activated caspase-9 and caspase-3 levels were significantly higher in the PTZ group (P<0.001, P<0.001), while pre-treatment with GSPE significantly decreased these levels compared to the PTZ group (P<0.001, P<0.001) (Fig. 7).

## Discussion

Although numerous advances have been made in the diagnosis and treatment of epilepsy, the precise pathogenic mechanism is obscure in the majority of patients, hampering effective treatment. In our study, GSPE pre-treatment partially suppressed seizures and reduced the PTZ-induced mortality rate. Of note, PTZ-induced kindling caused marked cognitive impairment as measured in the MWM, which is in agreement with our previous report (37), while GSPE pre-treatment had significant nootropic and neuroprotective effects, likely mediated by reduced oxidative stress and the rescue of mitochondrial function.

Chronic kindling by repeated chemical convulsant administration or electrical stimulation is among the most widely used models of chronic epilepsy for studies on epileptogenesis, novel drug targets, and treatment and preventative strategies (38). The underlying mechanisms of seizure pathogenesis have been proven to be complex and are not completely understood; several other molecular mechanisms are involved, including oxidative stress, inflammation, glutamate excitotoxicity and calcium overload. This is consistent with our results that GSPE pre-treatment partially suppressed, but not completely eliminated epileptic seizures. Moreover, in light of studies demonstrating that GSPE protects against oxidative stress (39) and enhances the working memory and cognitive performance in a rat model of Alzheimer’s disease (30), we primarily assessed the effects against epilepsy-associated oxidative stress and cognitive decline. As expected, PTZ-induced kindling caused an obvious increase in escape latency in MWM learning sessions and a marked decrease in time spent in the target quadrant during the probe (memory) trail. By contrast, pre-treatment with GSPE prior to PTZ significantly improved spatial learning and memory, as manifested by shorter escape latencies in learning sessions and longer time spent in the target quadrant during the probe trail. These results are consistent with those of a previous study (30).

Oxidative stress may be a critical pathogenic mechanism in the development of chronic epilepsy. Oxidative stress can be self-propagating in that initial oxidative damage creates additional free radicals, damages antioxidant enzymes, depletes antioxidant molecules, such as GSH and damages mitochondria, producing more ROS (11,40). Indeed, PTZ-induced seizures increased lipid peroxidation (MDA accumulation), decreased GSH in the hippocampus and enhanced ROS production in the mitochondria isolated from the epileptic hippocampus, implicating the exacerbation of oxidative stress in epileptic kindling (41). All these effects were partially or completely reversed by GSPE pre-treatment.

Mitochondria are the primary site of ROS production. Free radicals are highly reactive and thus damage biomolecules close to the site of generation, making mitochondria uniquely vulnerable to macromolecule dysfunction and DNA damage (12). Mitochondrial dysfunction and the overproduction of ROS may also enhance neuronal excitability and increase seizure susceptibility (42). Mitochondrial dysfunction is a common trigger for apoptosis, as ROS damage can induce Cyt *c* release and sequential activation of pro-apoptotic caspase-9 and caspase-3 (43,44). Therefore, synthetic antioxidants that protect mitochondrial targets and decrease neuronal death may be useful supplements for the clinical management of patients with seizures (45). Our results revealed that pre-treatment with GSPE decreased PTZ-induced mitochondrial ROS production and the degree of swelling and partially suppressed neuronal apoptosis, possibly through the protection of mitochondrial function and the inhibition of Cyt *c* translocation and caspase activation (46,47).

In order to further confirm the molecular change in the mitochondria that is responsible for the protective effects of GSPE pre-treatment, we focused on morphological observation. The findings from Nissl staining were consistent with the molecular results. Western blot analysis demonstrated that PTZ-induced seizures significantly increased the hippocampal expression of active caspase-3 and the release of cytosolic Cyt *c;* Nissl staining indicated significant pyramidal cell loss with ultrastructural signs of apoptosis. Furthermore, electron microscopy indicated that the mitochondria were damaged in the PTZ group, while GSPE pre-treatment reversed these morphological changes. Considering all of the above, targeting mitochondrial bioenergetics and oxidative stress with GSPE and non-pharmacological treatments may prove to be useful for the management of epilepsy (12).

In conclusion, GSPE, a safe and affordable intervention for clinical use, was effective in attenuating oxidative stress in the hippocampus of rats with chronic seizures. More importantly, pre-treatment with GSPE improved cognitive decline, at least in part, by protecting mitochondrial function and suppressing caspase-3-dependent apoptosis. However, due to the complex mechanisms of epilepsy, further studies are required to define the optimal treatment protocols and identify additional molecular targets of GSPE in epilepsy.
